# FK-3000 isolated from *Stephania delavayi* Diels. inhibits MDA-MB-231 cell proliferation by decreasing NF-κB phosphorylation and COX-2 expression

**DOI:** 10.3892/ijo.2015.2940

**Published:** 2015-03-27

**Authors:** HONG DE XU, SOON-CHANG CHO, MI-AE BANG, CHUN-SIK BAE, YEONSHIK CHOI, YONG-CHUN LI, SEUNG-KIL LIM, JAEGAL SHIM, DAE-HUN PARK

**Affiliations:** 1 School of Pharmaceutical Science, Zhengzhou University, Zhengzhou 450001, P.R. China; 2 NaturePureKorea Inc., Jeonnam 517-8-3, Republic of Korea; 3 Research Development Team, Jeonnam Biofood Technology Center, Jeonnam, Naju 520-330, Republic of Korea; 4 College of Veterinary Medicine, Chonnam National University, Gwangju 500-757, Republic of Korea; 5 Department of Bio Medical Analysis, Korea Polytechnic College, Nonsan, Chungnam 320-905, Republic of Korea; 6 Department of Exercise Prescription, Dongshin University, Naju, Jeonnam 520-714, Republic of Korea; 7 Comparative Biomedicine Research Branch, National Cancer Center, Goyang-si, Gyeonggi-do 411-769, Republic of Korea; 8 Department of Oriental Medicine Materials, Dongshin University, Naju, Jeonnam 520-714, Republic of Korea

**Keywords:** *S. delavayi* Diels., MDA-MB-231, 6,7-di-O-acetylsinococuline (FK-3000), apoptosis, NF-κB, COX-2

## Abstract

The World Health Organization (WHO) has reported that cancer is one of the most prevalent diseases and a leading cause of death worldwide. Many anticancer drug development studies have been pursued over the last few decades and several viable drugs have been discovered, such as paclitaxel, topotecan and irinotecan. Previously, our research group uncovered the cytocidal and cytostatic effects of the plant *Stephania delavayi* Diels. In this study, we determined the active chemical to be 6,7-di-O-acetylsinococuline (FK-3000). The FK-3000 half maximal inhibitory concentration (IC_50_) in MDA-MB-231 breast carcinoma cells at 48 h was 0.52 μg/ml and it induced apoptosis in a dose- and time-dependent manner. FK-3000 suppressed NF-κB nuclear translocation, decreased NF-κB phosphorylation, and decreased COX-2 protein expression. MDA-MB-231 xenografted mice were treated with FK-3000, Taxol, or their combination for 21 days. The tumor size was smallest in the co-treatment group, indicating that FK-3000 may have a synergistic effect with Taxol. FK-3000 treatment showed no adverse effects on blood cell counts, serum protein levels, or pathology. These studies demonstrate that FK-3000, isolated from *S. delavayi* Diels., is a promising, pathway-specific anticancer agent that exhibits low toxicity.

## Introduction

The World Health Organization (WHO) reported that in 2012 cancer was a leading cause of death with 8.2 million cancer deaths, 32.6 million cancer patients, and 14.1 million new cancer cases (1,2). In many cancers, the nuclear factor-κB (NF-κB) pathway is one of the most important for carcinogenesis, as its activation promotes tumor growth and progression (3,4). Inactive NF-κB is located in cytoplasm; however, when it is activated by phosphorylation it accumulates in the nucleus (5). Activated NF-κB transcription factor can inhibit apoptosis (6) and has been shown to upregulate expression of cyclooxygenase-2 (COX-2), a critical pro-survival inflammatory signaling molecule (7). In MDA-MB-231 breast cancer carcinoma cells, NF-κB and its inhibitor protein IκB are constitutively phosphorylated (8), which leads to chronic NF-κB activation and increased COX-2 expression (9).

Many non-specific inhibitors of NF-κB and the IκB kinase, IKKβ, have been developed and used to inhibit tumor growth and progression. These agents include anti-inflammatory drugs such as sulphasalazine and *trans*-resveratrol, non-steroidal anti-inflammatory drugs including aspirin, sulindac sulfide, cyclopentenone prostaglandins, and proteasome inhibitors, and glucocorticoids (10–12). COX-2 inhibitors have also been used successfully to slow cancer progression in patients (13,14). A selective COX-2 inhibitor, celecoxib, induces apoptosis by inactivating the pro-survival kinase Akt, both in the osteosarcoma cell line MG63 (15) and the liver cancer cell lines HepG2 and Hep3B (16). Another COX-2 inhibitor, NS-398, induces apoptosis in the colon carcinoma cell line HCA-7 (17) and promotes caspase-independent apoptosis in the hepatocellular carcinoma cell line Hep3B (18).

Epidemiological studies have demonstrated that a fruit and vegetable-rich diet reduces cancer incidence (19). Additionally, cancer morbidity is reduced by 50% when smoking cessation is combined with a low-fat diet rich in fruits and vegetables (20). Although few fruits and vegetables have been definitively shown to actively prevent or treat cancer, investigators continue to search for active agents in these food groups (21). Similarly, the anticancer benefits of herbal agents are due to their effects on signal transduction processes including NF-κB inhibition, apoptosis induction, DNA methylation, antioxidant activity, and metastasis inhibition. For example, lycopene in tomatoes exerts anticancer properties that are enhanced by vitamin E (22).

Many members of the *Stephania* plant family exhibit pharmacological benefits. For example, biscoclaurine alkaloid cepharanthine isolated from the herb *S. cepharantha* Hayata protects against DNA damage and scavenges free radicals to prevent lipid peroxidation (23). In addition, it induces G_0_/G_1_ cell cycle arrest and apoptosis by upregulating p15^INK4B^ and p21^Waf1/Cip1^ in 12PE myeloma cells (24). Bis-benzylisoquinoline alkaloid tetrandrine isolated from the roots of *S. tetrandra* S. Moore induces G_1_ arrest by downregulating E2F1 and upregulating p53/p21^Waf1/Cip1^ in human colon carcinoma HT29 cells (25). In 2011 our group reported that *S. delavayi* Diels. inhibits carcinoma proliferation (26), indicating that *S. delavayi* Diels. is a novel anticancer therapeutic candidate. This herb is already used in traditional Chinese medicine to relieve pain and cure acute gastroenteritis. However, the specific anticancer mechanism of action must be elucidated prior to its wide use in humans.

FK-3000, a component of the *S. delavayi* Diels. extract, has been reported to exhibit antiviral effects against herpes simplex virus type-1 (HSV-1) (27) and human immunodeficiency virus type 1 (HIV-1) (28,29). It also has been shown to downregulate NF-κB activity (30). Another extract constituent, sinococuline is an effective inhibitor of tumor cell growth (31) and exhibits antimalarial activity (33). Therefore, FK-3000 and sinococuline are prime candidates for the major active components in *S. delavayi* Diels.

In this study, we evaluated the anti-proliferative effect of 6,7-di-O-acetylsinococuline (FK-3000) isolated from *S. delavayi* Diels. against breast carcinoma associated with the apoptotic pathway via NF-κB and COX-2 *in vitro* and *in vivo*.

## Materials and methods

### Isolation of FK-3000 and sinococuline

**Figure 1:**
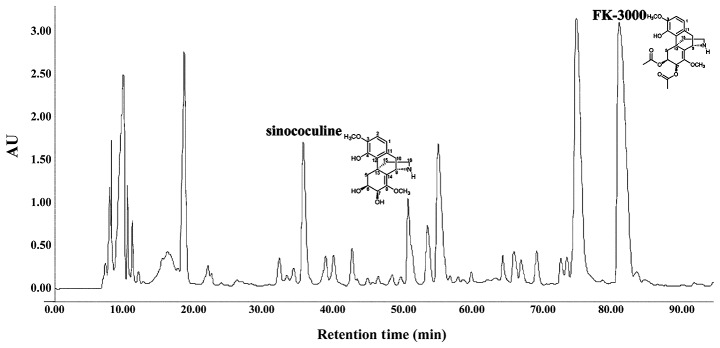
FK-3000 and sinococuline isolated from *S. delavayi* Diels. Fraction 3 from the initial *S. delavayi* Diels. extract chromatography was further purified by C18 HPLC. This yielded sinococuline at *R_t_* 36.01 min and FK-3000 at *R_t_* 82.14 min.

*S. delavayi* Diels. extract (1 g) was separated into 6 fractions by chromatography on a Sephadex LH-20 column with methanol (860×40 mm i.d., 25–100 μm). Fraction 3 (700 mg) was further purified by C18 high-performance liquid chromatography (HPLC) (YMC-Pack Pro, S-5 μm, 250×20 mm i.d.; 10–30% aqueous acetonitrile in 0.05% trifluoroacetic acid for 90 min at 7 ml/min), which yielded compound 1 (sinococuline) (15 mg, *R_t_* 36.01 min) and compound 2 (FK-3000) (76 mg, *R_t_* 82.14 min) (Fig. 1). The ^1^H, ^13^C, and two-dimensional nuclear magnetic resonance (2D NMR) spectra of the isolates were in good agreement with sinococuline and FK-3000 chemical structures (data not shown).

### Anti-proliferation evaluation

In order to evaluate the proliferation inhibition of *S. delavayi* Diels., sinococuline, and FK-3000, we used several cancer cell lines including MDA-MB-231 (human breast carcinoma), MCF-7 (human breast carcinoma), PC-3 (human prostate carcinoma), A-431 (human epidermoid carcinoma), HT-29 (human colorectal carcinoma), and CT-26 (murine colorectal carcinoma). These cell lines were obtained from the Korean Cell Line Bank (Seoul, Korea). Cells were seeded in triplicate into 96-well plates at a density of 1.5×10^4^ cells/well. Following a 12-h incubation, cells were treated with 0–16 μg/ml of *S. delavayi* Diels., 0–5 μg/ml of FK-3000, or 0–16 μg/ml of sinococuline. The control cells were treated with 0.1% DMSO alone. Following 48-h incubation, cell proliferation was analyzed using the CCK-8 cell counting kit (Dojindo Laboratories, Mashikimachi, Japan) according to the manufacturer’s instructions.

### Apoptosis induction analysis

MDA-MB-231 cells were seeded into 96-well plates as described above, incubated for 12 h, and treated with 0.5 or 5.0 μg/ml FK-3000. Following 48-h incubation, cells were harvested by trypsinization, washed in cold PBS, and resuspended in binding buffer (0.01 M HEPES/NaOH, 0.14 M NaCl, 2.5 mM CaCl_2_, pH 7.4). Annexin V-FITC (5 μl) (Becton-Dickinson, Franklin Lakes, NJ, USA) and 5 μl propidium iodide (Becton-Dickinson) were added to the cells followed by incubation with gentle mixing for 15 min at room temperature in the dark. Additional binding buffer was added and the Annexin V-stained cells were analyzed using a BD Model FACScan (Becton-Dickinson).

### Analysis of p-NF-κB localization

We measured activated NF-κB levels in MDA-MB-231 cells using an NF-κB translocation assay. Attached cells were treated with 5.0 μg/ml FK-3000 and incubated for 120 min in a Lab-Tek^®^ II Chamber Slide™ system (Nalge Nunc International). Cells were washed twice in cold PBS, fixed with cold acetone, blocked with Animal-Free Blocker™ (Vector, SP-5030) for 1 h, and incubated overnight at 4°C with a rabbit anti-human NF-κB p65 antibody (Cell Signaling, cat. no. 4764). Cells were incubated for 1 h with a FITC-conjugated anti-rabbit IgG (Cayman, cat. no. CAY-10006588), followed by DAPI staining. The cells were imaged using an IX51 Research Microscope (Olympus, Japan).

### Measurement of NF-κB phosphorylation and COX-2 expression levels

MDA-MB-231 cells were plated, incubated 12 h, then treated with 0.5 μg/ml or 5.0 μg/ml FK-3000. Following a 60-min to 48-h incubation, the cells were trypsinized, the harvested cells were washed twice with cold PBS, and total protein lysates were prepared using PRO-PREP™ (iNtRON Biotechnology, Seongnam, Korea) according to the manufacturer’s instructions. Cytosolic and nuclear proteins were separated using a Nuclear Extraction kit (Panomics, San Francisco, CA, USA) following the manufacturer’s protocol. The protein content of each sample was measured using the Bio-Rad Dc protein assay kit (Bio-Rad, Hercules, CA, USA) according to the manufacturer’s instructions. Equal protein amounts were loaded and separated on a 10% sodium dodecyl sulfate (SDS)-polyacrylamide gel, electrophoretically transferred to a nitrocellulose membrane using Trans-Blot^®^ Transfer Medium (Bio-Rad), and incubated with the following antibodies: monoclonal mouse anti-human *p*-NF-κB antibody (Cell Signaling, cat. no. 3036, Danvers, MA, USA), polyclonal mouse anti-human COX-2 antibody (Cayman, cat. no. CAY-160106, Ann Arbor, MI, USA), monoclonal β-actin antibody (Sigma-Aldrich, Inc., cat. no. A-5316, St. Louis, MO, USA), or monoclonal PARP antibody (Biomol International, cat. no. SA-250, Plymouth Meeting, PA, USA). HRP-conjugated goat anti-rabbit IgG (Cayman, cat. no. 10004301) and antimouse IgG (Cell Signaling, cat. no. 7076) were used as secondary antibodies. The bands were visualized using an ECL detection kit (Amersham Biosciences, UK) according to the manufacturer’s protocol and a LAS 3000 imaging system (Fuji Film, Japan).

### Assessment of tumor growth

The human tumor xenograft study was approved by the Institute of Animal Care and Use Committee prior to performing the experiments. Forty, 8-week-old, female BALB/cnu/nu mice were purchased from OrientBio (Sungnam, Korea) and allowed to acclimate for 7 days. All animals were housed in a temperature and relative humidity-controlled environment (22±3°C, 50±5%, 12-h light/dark cycle) throughout the acclimation and experimental period. The mice were provided a Purina diet (Purina Korea) and water *ad libitum*. Mice were subcutaneously injected with 5×10^6^ of MDA-MB-231 cells in each flank. When the tumor volumes reached 100–150 mm^3^, mice were randomly divided into four groups. The first group (control group, n=7) was intraperitoneally administered vehicle (0.1% DMSO, once a day). The second, third, and fourth groups (n=8 each) received Taxol (Sigma-Aldrich, 10 mg/kg body weight, intraperitoneally once per week), FK-3000 (1 mg/kg body weight, intraperitoneally daily), or Taxol (10 mg/kg, intraperitoneally once a week) and FK-3000 (1 mg/kg, intraperitoneally daily) for 24 days. The tumors were measured by caliper every 3 days and tumor volumes were calculated using *axb^2^/2* (where *a* was the width at the widest tumor point and *b* was the width perpendicular to *a*). The mice were sacrificed at day 25.

### Histopathological examination

After all the animals were sacrificed, organ weight was measured of brain, pituitary gland, liver, spleen, heart, thymus, salivary gland, kidney, adrenal gland, lung, thyroid gland/parathyroid gland, seminal vesicle (male only), prostate (male only), testes (male only), epididymis (male only), ovary (female only), and uterus/cervix (female only). Histopatholgical examination was conducted only in the control group (0 mg/kg/day PbL treatment group) and the high dosing group (3,000 mg/kg/day treated group) Testes and epididymis were fixed with Bouin solution and the other tissues were fixed in 10% (v/v) formaldehyde solution, dehydrated with ethanol (99.9, 90, 80 and 70%) and water, and embedded in paraffin. Specimens were sliced into sections of 5-μm thickness. The slides were stained with hematoxylin and eosin (H&E).

### Statistical analysis

Results are expressed as mean ± standard deviation (SD). Groups were compared using Tukey’s studentized range (HSD) test with SPSS Statics (IBM, Armonk, NY, USA); Statistical significance ^*^p<0.1; ^**^p<0.05.

## Results

### FK-3000 has an anti-proliferative effect against several carcinomas

We chromatographically isolated two structurally similar compounds from *S. delavayi* Diels. extract. The ^1^H, ^13^C and 2D NMR spectra of these compounds were consistent with those published previously for FK-3000 and sinococuline (Fig. 1) (33,34). We compared the inhibitory effects of *S. delavayi* Diels. extract, sinococuline, and FK-3000 on proliferation in several cancer cell lines (Table I). FK-3000 more effectively inhibited cell proliferation in the six carcinomas tested when compared to *S. delavayi* Diels. extract or sinococuline. In particular, MDA-MB-231, MCF-7, PC-3, and HT-29 cell growth was more sensitive to FK-3000. Sinococuline was less effective than *S. delavayi* Diels. extract at inhibiting growth in the six cancer lines tested. In MDA-MB-231 cells at 48 h post-treatment, the IC_50_ ranges of *S. delavayi* Diels. extract, sinococuline, and FK-3000 were 1.20–5.32, 4.49–15.88 and 0.22–2.70 μg/ml, respectively.

### FK-3000 increases MDA-MB-231 cell apoptosis in a dose-and time-dependent manner

**Figure 2:**
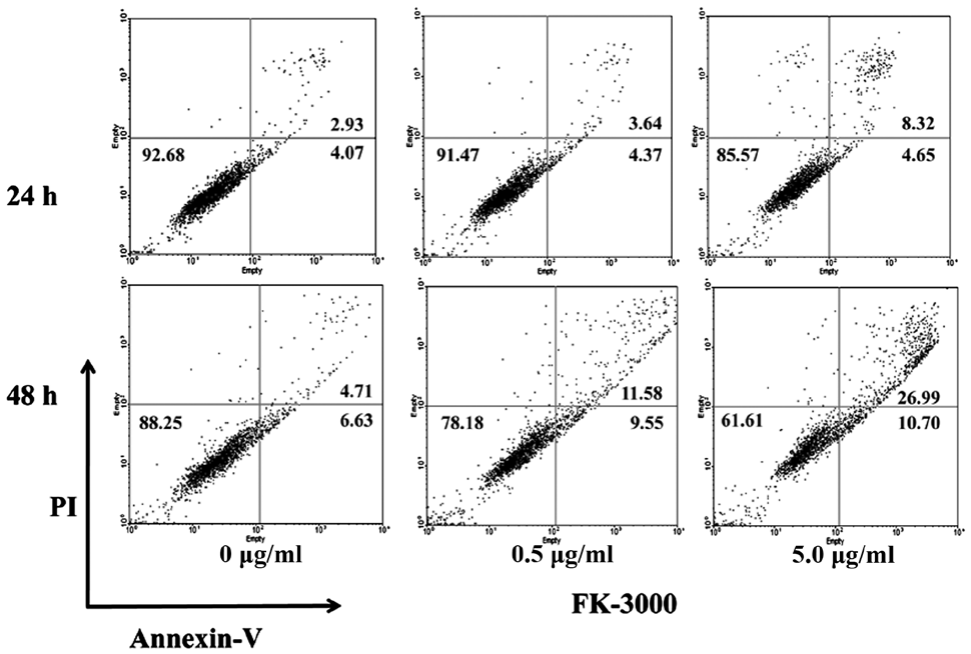
FK-3000 increased MDA-MB-231 cell apoptosis in a dose- and time-dependent manner. In the 5.0 μg/ml FK-3000-treated cells, the apoptotic cell percentage increased from 12.97% at 24 h to 37.69% at 48 h. Following 48-h incubation, the percentage of apoptotic cells was 11.34% in control cells and 37.69% in 5.0 μg/ml FK-3000-treated cells.

FACS analysis demonstrated that FK-3000 induced apoptosis in a dose- and time-dependent manner (Fig. 2). After 24-h treatment with 0.5 μg/ml FK-3000, the percentage of apoptotic cells was ~8.01% and by 48 h it had increased to 21.13%, compared to 7.00 and 11.34%, respectively, in vehicle-treated cells. At 5.0 μg/ml FK-3000 dosage, the percent of apoptotic cells increased to 12.97% after 24 h and 37.69% at 48 h.

### FK-3000 effectively blocks NF-κB nuclear translocation

**Figure 3:**
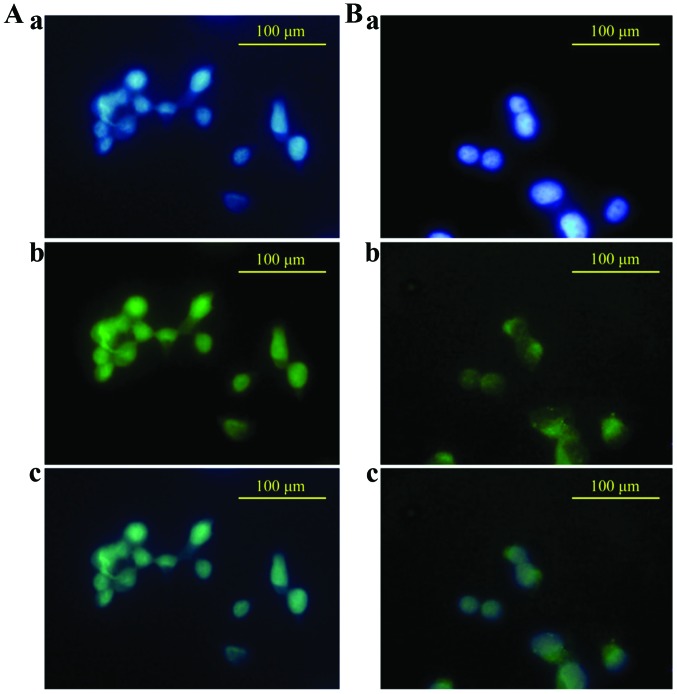
FK-3000 effectively blocks NF-κB translocation from cytoplasm to nucleus. (A) In untreated MDA-MB-231 cells NF-κB p65 proteins [green color (b)] are localized primarily in the nucleus [blue color (a)]. The merged image (c) confirms the NF-κB p65 protein localization. (B) In the 5.0 μg/ml FK-3000-treated cells, NF-κB p65 proteins (b) were almost exclusively localized to the cytoplasm (a). The merged image (c) confirms that the NF-κB p65 proteins are not in the cell nucleus.

In most cancer cells NF-κB proteins are active and localized to the nucleus, which inhibits apoptosis induction. This is in contrast to normal cells where it is localized in the cytoplasm in an inactive form (36). To confirm that FK-3000 inactivates NF-κB, we evaluated whether it blocks NF-κB nuclear localization (Fig. 3). Untreated MDA-MB-231 cell staining clearly showed that NF-κB p65 proteins were primarily localized to the nucleus (Fig. 3A-c). In the FK-3000 treated cells NF-κB p65 proteins were localized mainly to the cytoplasm (Fig. 3B-c) This demonstrates that FK-3000 effectively inhibited NF-κB translocation from the cytoplasm to nucleus.

### FK-3000 decreases NF-κB phosphorylation and COX-2 protein expression

**Figure 4:**
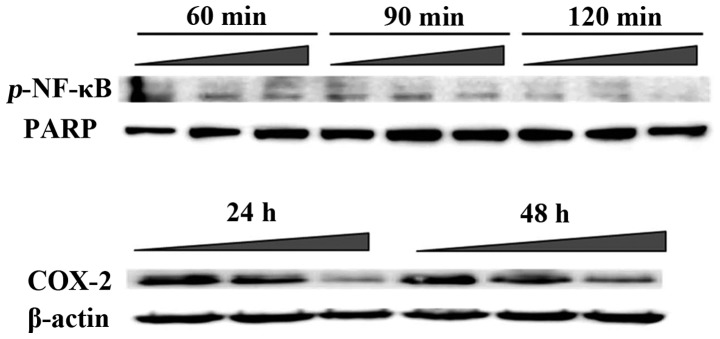
FK-3000 reduced NF-κB phosphorylation levels and COX-2 expression. In MDA-MB-231 cells treated with 5.0 μg/ml FK-3000 for 120 min, NF-κB phosphorylation decreased to nearly undetectable levels compared with the 0.5 μg/ml treated cells at 120 min or the 5.0 μg/ml treated cells at 60–90 min. FK-3000 suppressed COX-2 protein expression in a dose- and time-dependent manner.

There are several mechanisms to induce cell apoptosis including the caspase cascade, the Bcl family pathway (35), or the NF-κB-COX-2 pathway (36). In our previous study on *S. delavayi* Diels., the FK-3000 parental material demonstrated an anti-proliferative effect via the NF-κB-COX-2 pathway (26). Therefore, we investigated whether FK-3000 could induce apoptosis in the same manner using MDA-MB-231 cells. In order to determine FK-3000’s NF-κB phosphorylation (activation) inhibitive effect, we performed western blot analyses of the p-NF-κB levels at various time points from 60–120 min following 5.0 μg/ml FK-3000 treatment (Fig. 4). The phosphorylation of NF-κB decreased in a time-dependent manner and by 120 min was nearly undetectable. Therefore, FK-3000 effectively suppressed the phosphorylation of NF-κB. COX-2 protein can induce apoptosis in cells and its expression is controlled by NF-κB (36). At 24 h following FK-3000 treatment, the COX-2 protein level decreased in a dose-dependent manner (Fig. 4). At 48 h post-treatment the result was unchanged.

### FK-3000 inhibits cancer cell growth in a mouse xenograft model

**Figure 5:**
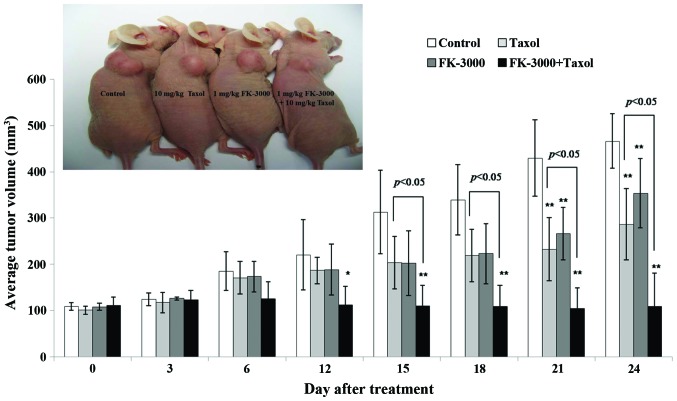
FK-3000 inhibits tumor growth in an MDA-MB-231 xenografted mouse model. Tumor sizes in mice treated with vehicle alone (0.1% DMSO), FK-3000 (1 mg/kg body weight/day), Taxol (10 mg/kg body weight/week), or FK-3000 and Taxol combined. From day 15 onwards, tumor volumes of the drug-treated groups were significantly different from the control. Values represent the mean ± standard deviation. ^*^p<0.1; ^**^p<0.05, compared to the control, as determined by Tukey’s range (HSD) test.

To assess whether FK-3000 is a viable candidate for anticancer therapy, we used an MDA-MB-231 xenografted mouse model to directly evaluate its antitumor effects (Fig. 5). Mice were treated with vehicle, FK-3000, Taxol, or FK-3000 in combination with Taxol. At 12 days of treatment, the tumor volume in the Taxol and FK-3000 co-treatment group was the smallest among the four groups (p<0.05). Following 21 days of treatment, tumor volumes were significantly different in all the treatment groups compared to the control (p<0.05). FK-3000 alone inhibited tumor growth to a similar extent as Taxol. Additionally, FK-3000 treatment showed no signs of toxicity. There were no differences in liver function tests, complete blood cell counts, and serum enzyme levels between any of the drug treatment groups and the controls (Tables I and II). There were no histopathological changes observed in any group (data not shown). Interestingly, FK-3000 and Taxol co-treatment exhibited a synergistic effect (Fig. 5).

## Discussion

Previously, we reported that *S. delavayi* Diels. suppressed MDA-MB-231 carcinoma proliferation by inducing apoptosis (26). In this study FK-3000 and sinococuline were isolated from *S. delavayi* Diels. extract (Fig. 1). FK-3000 and sinococuline inhibited proliferation in several carcinomas including MDA-MB-231, MCF-7, PC-3, A-431, HT-29, and CT-26 (Table I). The anti-proliferative effect was greatest using FK-3000, followed by *S. delavayi* Diels. extract, and sinococuline. FK-3000 induced dose- and time-dependent apoptosis in MDA-MB-231 cells and the 5.0 μg/ml FK-3000 treatment increased the percentage of apoptotic cells from 12.97% at 24 h to 37.69% at 48 h, a 26.35% increase compared to control cells (Fig. 2). The active form of NF-κB is phosphorylated and localized to the nucleus. In MDA-MB-231 cells NF-κB is constitutively active (6). FK-3000 at a 5.0 μg/ml dose significantly blocked NF-κB translocation from the cytoplasm to the nucleus (Fig. 3). FK-3000 inhibited both NF-κB phosphorylation and COX-2 protein expression in a dose- and time-dependent manner (Fig. 4). At 120 min with 5.0 μg/ml FK-3000, the NF-κB was almost completely dephosphorylated. FK-3000 inhibited tumor growth in the MDA-MB-231 xenograft model. FK-3000 is as effective as Taxol, with daily 1 mg/kg body weight FK-3000 treatments exhibiting similar effects to weekly 10 mg/kg body weight Taxol administration. Since an overall lower FK-3000 dose (7 mg/kg body weight/week) was able to reduce tumor growth to the same degree as Taxol (10 mg/kg body weight/week), FK-3000 may be a more effective antitumor agent. FK-3000 also had a synergistic effect when used in combination with Taxol (Fig. 5). This may be due to modulation of different pathways, with FK-3000 targeting NF-κB activation and Taxol blocking cell mitosis (37). As a whole, these observations suggest that FK-3000 is a promising anticancer drug candidate.

Many epidemiological studies report that vegetable-rich diets reduce both cancer incidence and morbidity, but the action mechanisms are ambiguous in most cases. Thus, identifying the specific antitumor effect mechanisms of a plant is an active area of research. For example, Nexrutine, a *Phellodendron amurense* herbal extract, has been investigated as a prostate cancer treatment (38,39). In this study we determined that the apoptosis induction effect seen with *S. delavayi* Diels. extract is caused by its active compound FK-3000 through NF-κB deactivation. NF-κB activation is a double-edged sword and its downstream effects depend on the cell’s phenotype and context.

NF-κB activation inhibits apoptosis (40,41) by altering the apoptosis related protein 3 (APR3) levels that normally change during development and inflammation (42). It also suppresses TNF-α-induced apoptosis by promoting transcription of apoptotic inhibitors such as Bcl-2, inhibitor of apoptosis proteins (IAPs), and TNFR-associated factors (TRAF) 1 and 2 (43–45). Conversely, NF-κB activation can also promote apoptosis, as evidenced by doxorubicin-mediated cell death induction through IκB degradation in N-type neuroblastoma cells (46) and p53-induced apoptosis, which depends on NF-κB activation (47). In the case of breast carcinomas, constitutive NF-κB activation is detrimental to the patient prognosis, and treatment with a compound like FK-3000 could potentially improve outcomes.

Furthermore, we tested the safety and efficacy of FK-3000 in a mouse xenograft model. We identified several advantages to developing FK-3000 as a novel anticancer drug. First, FK-3000 seems to be very safe with low toxicity (Tables II and III) compared to other small molecule inhibitors. For example, the COX-2 inhibitor celecoxib has numerous side effects including gastric bleeding (48). Second, FK-3000 specifically targets the NF-κB and COX-2 pathway. Third, since Taxol is a major anticancer drug approved to treat several types of cancer; combination of FK-3000 and Taxol may improve the outcome of anticancer chemotherapy. Overall, FK-3000 is a promising candidate to inhibit cancer proliferation.

## Figures and Tables

**Table I:** The half maximal inhibitory concentration (IC_50_) of *S. delavayi* Diels., sinococuline and FK-3000 on six carcinoma cell lines at 48 h.

Cell line	*S. delavayi* Diels. (μg/ml)	Sinococuline (μg/ml)	FK-3000 (μg/ml)
MDA-MB-231	2.31	4.49	0.52
MCF-7	2.05	14.45	0.77
PC-3	1.20	6.81	0.22
A-431	5.32	6.83	2.70
HT-29	4.57	15.88	0.40
CT-26	3.37	11.21	1.90
Average	3.137	9.945	1.085

**Table II:** Complete blood cell counts from MDA-MB-231 xenografted mice in the treatment and control groups.

	Control	Taxol	FK-3000	FK-3000+Taxol
Leukocytes				
WBC (k/μl)	3.39±2.54	7.12±3.17	5.06±2.56	3.93±1.47
NE (k/μl)	1.12±0.78	2.24±1.05	1.99±1.11	1.55±0.95
LY (k/μl)	1.89±1.55	4.03±1.76	2.41±1.29	2.01±0.45
MO (k/μl)	0.17±0.13	0.42±0.18	0.29±0.12	0.20±0.07
EO (k/μl)	0.16±0.13	0.33±0.18	0.29±0.13	0.15±0.10
BA (k/μl)	0.05±0.03	0.10±0.05	0.09±0.05	0.05±0.05
Erythrocytes				
RBC (M/μl)	9.36±0.41	9.47±0.42	9.53±1.16	9.57±0.31
Hb (M/dl)	12.76±0.42	13.14±0.49	10.80±5.13	13.30±0.63
HCT (%)	49.84±2.22	50.16±1.36	50.86±4.94	52.00±1.52
MCV (fl)	53.26±0.91	53.54±1.76	53.54±1.76	54.34±1.69
MCH (pg)	13.66±0.49	13.56±1.31	13.56±1.31	13.88±0.51
MCHC (g/dl)	25.62±0.76	25.26±1.71	25.26±1.71	25.58±0.91
RDW (%)	16.46±0.43	17.42±2.30	16.88±1.70	16.80±0.25
Thrombocyte				
PLT (k/μl)	521.8±142.7	392.6±57.36	234.8±73.58	435.0±166.2
MPV (fl)	4.74±0.17	5.10±0.60	5.36±0.60	4.92±0.40

Each group was administered vehicle (0.1% DMSO), Taxol (10 mg/kg body weight/week), FK-3000 (1 mg/kg body weight/day), or both Taxol and FK-3000. There was no difference in the blood cell counts between the groups.

**Table III:** Complete blood chemistry from MDA-MB-231 xenografted mice in the treatment and control groups.

	Control	Taxol	FK-3000	FK-3000+Taxol
GOT (U/I)	72.2±10.63^a^	63.60±3.71	65.20±3.77	64.40±4.50
GPT (U/I)	25.00±2.55^a^	30.20±5.20	25.80±2.17	24.60±2.61
BUN (mg/dl)	42.84±9.47^a^	36.50±4.45	52.00±6.32	59.30±11.58
NH_3_ (μg/dl)	95.4±3.51^a^	97.80±5.07	92.00±1.41	97.40±3.78
TBIL (mg/dl)	0.36±0.15^a^	0.38±0.13	0.44±0.09	0.43±0.18
ALB (g/dl)	2.34±0.15^a^	2.32±0.22	2.46±0.15	2.42±0.13

Each group was administered vehicle (0.1% DMSO), Taxol (10 mg/kg body weight/week), FK-3000 (1 mg/kg body weight/day), or both Taxol and FK-3000.

a The differences are statistically not significant between each group.
